# Aortic Origins of the Celiac Trunk and Superior Mesenteric Artery

**DOI:** 10.3390/diagnostics11061111

**Published:** 2021-06-18

**Authors:** Mugurel Constantin Rusu, Adelina Maria Jianu, Bogdan Adrian Manta, Sorin Hostiuc

**Affiliations:** 1 Division of Anatomy, Department 1, Faculty of Dental Medicine, “Carol Davila” University of Medicine and Pharmacy, RO-020021 Bucharest, Romania; bogdan.manta@drd.umfcd.ro; 2 Department of Anatomy and Embryology, Faculty of Medicine, “Victor Babeș” University of Medicine and Pharmacy, RO-300041 Timişoara, Romania; adelina.jianu@gmail.com; 3 Department of Legal Medicine and Bioethics, Faculty of Dental Medicine, “Carol Davila” University of Medicine and Pharmacy, RO-020021 Bucharest, Romania

**Keywords:** aorta, computed tomography, vertebral column, celiac-mesenteric axis, anatomic variation

## Abstract

(1) Background. The vertebral level of origin (VLO) of the celiac trunk (CT) and superior mesenteric artery (SMA) has been scarcely investigated. (2) Method. This study used 107 computed tomography angiograms and an eleven type grading system to classify the VLO of the CT and SMA. Each of the T12–L2 vertebra were divided in three horizontal levels. The intervertebral discs were considered distinct levels. (3) Results. The VLO of the CT ranged from the upper third of the T12 vertebra to the lower third of the L1 vertebra. The VLO of the SMA ranged from the lower third of the T12 vertebra to the upper third of the L2 vertebra. There was a highly significant association between the VLO of the CT and SMA (Chi2 = 201, *p* < 0.001), usually respecting a “plus two” rule. The mean CT–SMA distance was 1.82 +/− 0.66 cm in males and 1.55 +/− 0.411 cm in females, the difference being statistically significant. The mean CT–SMA distance tended to decrease with increasing CT–SMA types, the differences being statistically significant. (4) Conclusions. These characteristics of CT and SMA origins and their relations should be known by surgeons, as they could impact operative management and should be evaluated on a case-by-case basis.

## 1. Introduction

The vertebral level of origin of the celiac trunk (CT) and the superior mesenteric artery (SMA) has been scarcely investigated. As Anson and McVay wrote in 1936, “the regular textbooks of gross anatomy offer little precise information concerning the points at which the visceral branches of the abdominal aorta arise, either in relation to the vertebrae or the bifurcation or in relation to one another” [1]. We found only a few studies documenting the possibilities of variation of these vertebral levels [1,2,3,4,5,6,7,8,9]. The authors used a vertical grading system considering the intervertebral discs and the upper, middle, and the lower third of each vertebra, respectively [4,5]. However, the authors did not evaluate the combined patterns of the CT and SMA vertebral origins. We, therefore, hypothesized that, as the roots of the two aortic branches are one above the other, the vertebral levels of the roots might be correlated. To test this hypothesis, we performed a topographic and morphometric analysis of the aortic origins of the CT and SMA on computed tomography (CT) angiograms to check whether or not the levels of origin of the CT and SMA correlate significantly.

## 2. Materials and Methods

### 2.1. Study Group

We conducted a retrospective study on 114 randomly selected computed tomography angiograms to evaluate the CT and SMA’s aortic origins. Inclusion criteria were the age of the subjects (>18 years), adequate quality of the angiograms, and no previous history of surgery on abdominal vessels. Exclusion criteria were pathological processes distorting the arterial anatomy, degraded or incomplete computed tomography scans [10], no prior known vertebral column abnormalities (including kyphosis, scoliosis or other vertebral pathology), and no vertebral column surgery. After applying these criteria, we retained 107 angiograms from 68 male and 39 female subjects aged between 54- and 71-years-old. Subjects were of Caucasian (Romanian) origin. All subjects gave their informed consent for inclusion before they participated in the study. The research was conducted following principles from The Code of Ethics of the World Medical Association (Declaration of Helsinki). The responsible authorities (affiliation of the 2^nd^ author) approved the study (approval no.37/28.08.2020).

The computed tomographic exams consisted of injecting an iodine radiocontrast agent in the brachial vein, followed by iodine radiocontrast agent and saline medium. The computed tomography scan was performed using a 32-slice scanner (Siemens Multislice Perspective Scanner, Forchheim, Germany), with 0.6 mm collimation and reconstruction of 0.75 mm thickness with 50% overlap for multiplanar, MIP, and 3D Volume-Rendering technique [11]. The specific arterial anatomy was documented using the Horos Project software for iOS and its 3D Volume Rendering application. Two authors did the measurements.

### 2.2. Definition of Variables

**Figure 1:**
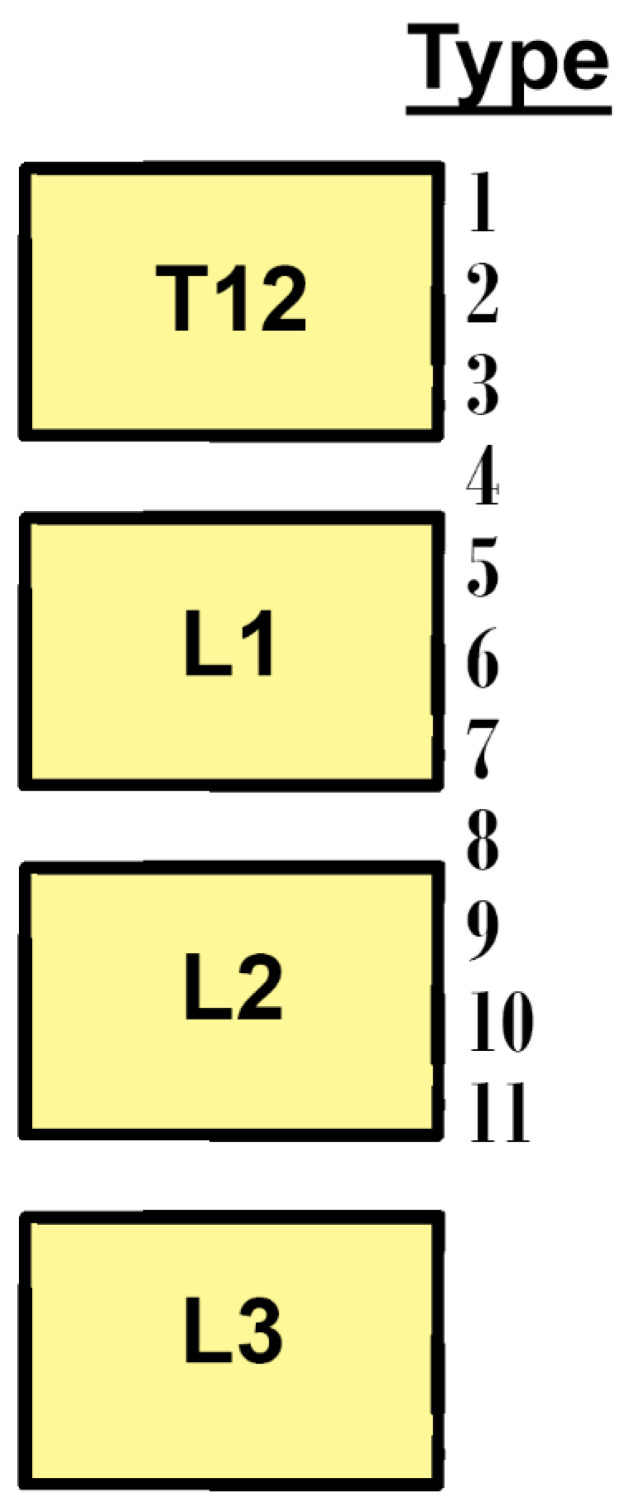
Vertebral levels were used to grade the topography of the aortic origins of the celiac trunk and superior mesenteric artery.

Grading systems were used to classify the vertebral levels for the CT and SMA’s aortic origins (Figure 1). The height of a vertebra corresponded to three levels; superior, middle, and inferior. The intervertebral discs were considered distinct vertebral levels.

### 2.3. Statistical Tests

For the statistical tests were used Jamovi for MacOS [12,13,14,15]. We used Cohen’s kappa to test interrater reliability, Chi^2^ to test the association between gender and anatomical variables, and between CT and SMA types. We used ANOVA to test the presence of differences between mean CT–SMA distances depending on sex and type.

## 3. Results

### 3.1. Prevalence of Types

The vertebral level of the aortic origin of CT was type 1 in 7/107 cases (6.54%), type 2 in 10/107 patients (9.34%), type 3 in 20/107 patients (18.69%), type 4 in 21/107 cases (19.63%), type 5 in 26/107 cases (24.3%), type 6 in 16/107 patients (14.95%), and type 7 in 7/107 cases (6.54%).

The vertebral level of the aortic origin of SMA was type 3 in 3/107 cases (2.8%), type 4 in 11/107 patients (10.28%), type 5 in 12/107 patients (11.21%), type 6 in 27/107 cases (25.23%), type 7 in 35/107 patients (32.71%), type 8 in 14/107 patients (13.08%), and type 9 in 5/107 cases (4.67%).

The distribution of types for each gender is shown in Table 1.

**Figure 2:**
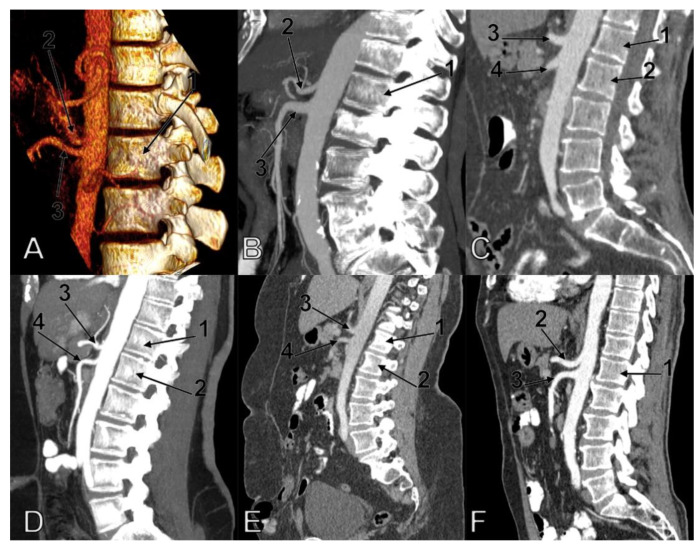
Combined patterns of the vertebral levels (types) of the aortic origins of the celiac trunk (CT) and superior mesenteric artery (SMA). (**A**) Three-dimensional volume rendering of a 5/6 type combination, left lateral view (1.1st lumbar vertebra; 2.CT; 3.SMA). (**B**) Sagittal slice of a 6/9 type combination (1.1st lumbar vertebra; 2.CT; 3.SMA). (**C**) Sagittal slice of a 7/9 type combination (1.1st lumbar vertebra; 2nd lumbar vertebra; 3.CT; 4.SMA). (**D**) Sagittal slice of a 4/6 type combination (1.12th thoracic vertebra; 2.1st lumbar vertebra; 3.CT; 4.SMA). (**E**) Sagittal slice of a 6/8 types combination (1.1st lumbar vertebra; 2.2nd lumbar vertebra; 3.CT; 4.SMA). (**F**) Sagittal slice of a 5/7 types combination (1.1st lumbar vertebra; 2.CT; 3.SMA).

The combinations of CT and SMA types were recorded in the general lot (*n* = 107) and for each gender (Table 2). The following varieties of types (CT/SMA) were not found: 1/2, 1/5, 1/8, 1/9. 2/3, 2/8, 2/9, 3/8, 3/9, 4/9, 5/9, 7/7. Overall, the 5/7 pattern prevailed (17.57%). The 1/4, 1/6, 2/5, 6/9 combinations of types were rare, occurring each in 0.93% of cases. In males, the combinations 2/5, 7/7, 3/7, and 4/5 were not found, while in females the combinations 1/4, 1/6, 2/5, 6/9, 7/7, 1/7 were not found. The 5/7 pattern prevailed in both males (10.11%) and females (15.38%). Different samples of combinations of the vertebral levels of the CT and SMA are presented in Figure 2.

### 3.2. Statistical Analysis

Cohen’s kappa test had a value of 0.953 (*p <* 0.001), suggesting the presence of high interrater reliability regarding the assessment of anatomical variations.

Most males had CT type 5 (18 cases, 26.5%), followed by type 6 (13 cases, 19.1%) and type 3 (12 cases, 17.6%). Most females had CT type 4 (11 cases, 28.2%), followed by type 5 (9 cases, 23.1%) and type 3 (8 cases, 20.5%). See Table 3 for details. There were no statistically significant differences in the CT type depending on the sex of the subject (Chi^2^ *=* 4.95, *p* = 0.55).

Most males had SMA type 7 (24 cases, 35.3%), followed by type 6 (14 patients, 20.6%) and types 4 and 8 (10 cases, 14.7%). Most females had SMA type 6 (12 patients, 30.8%), followed by type 7 (10 cases, 25.7%) and type 5 (7 cases, 18%). See Table 4 for details. There were no statistically significant differences in the SMA type depending on the sex of the subject (Chi^2^ *=* 7.42, *p* = 0.285).

We found a highly significant association between CT and SMA types (Chi^2^ = 201, *p <* 0.001), usually respecting a “plus two rule”—for example, most type 3 SMA were associated with type 1 CT, most type 6 SMA were associated with type 4 CT, and so on. See Table 5 for details.

**Figure 3:**
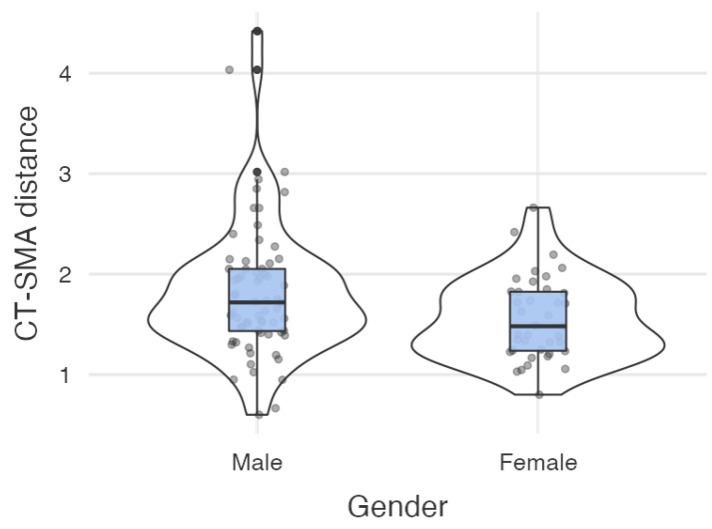
CT–SMA distance depending on gender.

The mean CT–SMA distance was 1.82 +/− 0.66 cm in males and 1.55 +/− 0.411 cm in females. The difference was statistically significant (ANOVA, F *=* 6.53, *p* = 0.012). See Figure 3 for details.

**Figure 4:**
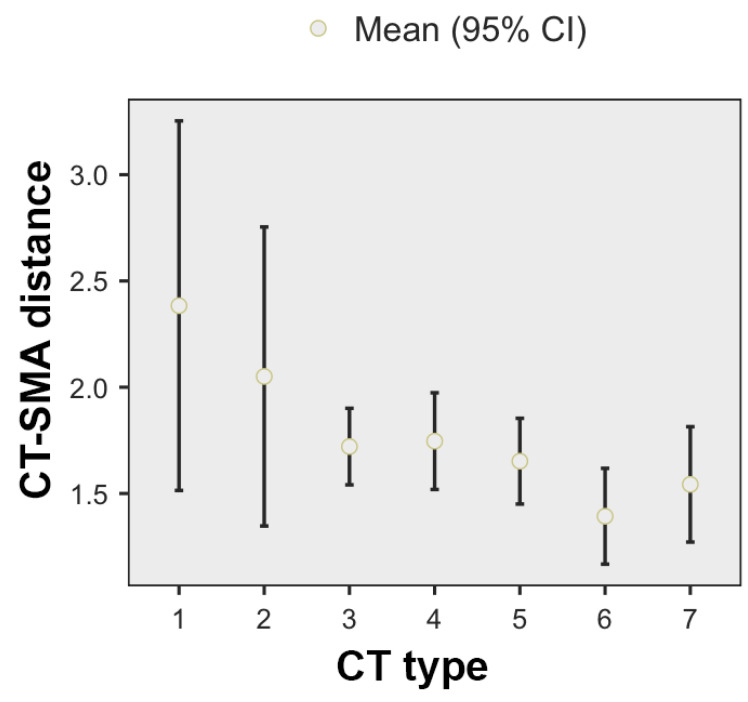
CT–SMA distance, depending on the CT type.

When using the origin of the CT as a reference, the highest mean CT–SMA distance was found for type 1 CT (2.38 +/− 0.94 cm), while the lowest for type 6 CT (1.39 +/− 0.423). The mean CT–SMA distance tends to decrease with increasing CT–SMA types, being identifiable three main groups: the first one includes the first two CT types, in which the average CT–SMA distance is above 2 cm, the second group contains the third, fourth and fifth CT types, in which the average CT–SMA distance is around 1.7 cm, and the third group, which includes the sixth and seventh CT types, in which the average CT–SMA distance is approximately 1.45 cm. See Figure 4 for details. The differences were statistically significant (ANOVA, F *=* 2.14, *p* = 0.078).

**Figure 5:**
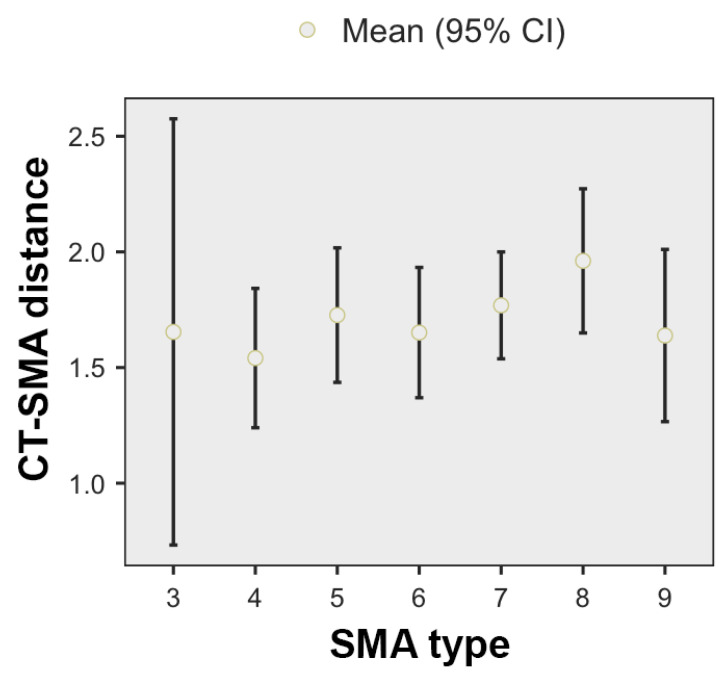
CT–SMA distance, depending on the SMA type.

When using the type of SMA as a reference (Figure 5), the highest mean CT–SMA distance was found for type 8 CT (1.96 +/− 0.54 cm), while the lowest for type 4 SMA (1.54 +/− 0.5). When analyzed in correlation with the SMA type, the CT–SMA distance did not differ significantly depending on the variant (ANOVA, F *=* 0.757, *p* = 0.612).

## 4. Discussion

Numerous studies evaluated the CT and SMA branching patterns [16,17,18,19,20,21,22], with little emphasis on their origin relating to vertebral levels. However, the abdominal aorta’s topography and its branches, related to the vertebral column, are surgically significant [23]. When a main artery, CT or SMA, switches its vertical position as referred to the vertebral column, all its branches could have modified topographical patterns, which various branching patterns could complicate. Different procedures, such as the anterior access to the lumbar spine, lymphadenectomy of the splanchnic lymph nodes, and celiac plexus block, rely on the adequate identification of arterial–vertebral topographic patterns [23]. The surgical fixation of lumbar spine instability using anterior lumbar interbody fusion gained popularity after the introduction of minimally invasive surgical techniques, mini-laparotomic or endoscopic approaches [24]. However, these procedures are associated with perioperative vascular complications [24]. A good knowledge of vertical topography and significant patterns, such as the “plus two” rule of the aortic origins of the abdominal aorta anterior branches that we found here, might aid in avoiding arterial damages during robotic and laparoscopic surgery, as previously discussed [5]. This is equally important for interventional radiologists during catheterizations [5], as well as for vascular surgeons, oncologists, and anatomists [25]. Nevertheless, the CT and SMA could be iatrogenically injured during approaches of different neighbor tumors and extensive adenopathies, such as left radical nephrectomies [26]. In these cases, surgeons should not assume a specific pattern of the vertebral level of arterial origins and should document the case on CT angiograms for a personalized approach.

Gregory et al. found that in children, the most frequent vertebral levels of aortic origins for the CT and SMA were T12 and L1, respectively. Still, for each vessel, they found significant variation of the vertebral level [23]. They also found an association between CT vertebral level and gender, with males tending to have a more substantial percentage of CTs (23.1%) arising at the level of the first lumbar vertebra compared to females (9.2%) [23]. They failed to show a potential association between the SMA vertebral level origin and gender [23].

Matusz documented a few grading studies regarding the vertebral level of origin of the CT and SMA [25] that were published by Adachi (1928), Ruggles (1935), and Cauldwell and Anson (1943) [2,3,27]. The authors [2,4,5,25] cited Ruggles’ study using the first name of the author (“George R”).

Ruggles, and Cauldwell and Anson, found that in most cases the origin of the CT corresponds to the upper third of L1 (25/97 cases of Ruggles’ dissection study, 75/300 cases of Cauldwell and Anson’s dissection study) and the origin of the SMA corresponds to the lower third of L1 (29/97 cases of Ruggles’ study, 67/300 cases of Cauldwell and Anson’s study), a result confirmed by us. According to Matusz, Adachi found the most frequent origin of the CT was at the intervertebral disc between T12 and L1 (15/48 cases) and the origin of SMA at the level of the upper third of L1 (12/47 cases). However, Ruggles also documented Adachi’s works and reported that the mean topographical pattern for the CT was found by Adachi in 50 cases to be in the upper third of the first lumbar vertebra, while the mean pattern for the SMA was according to Adachi, was at the middle third of L1 [3]. Anson and McVay (1936) conducted an anatomical study of the CT and SMA [1]. The CTs originated in 74% at the first lumbar vertebra while the SMA originated in 83% in an area from the middle third of the first lumbar vertebra (type 6 in this study) to the upper third of the second lumbar vertebra (type 9 in this study) [1]. They also reported that in 71% of cases, the distance between the aortic origins of the CT and SMA was between 1 cm and 2 cm, without any other details [1]. They also found that “there is no regular association between the length of the aorta and the relation of the visceral branches to the vertebrae“ [1]. Panagouli et al. (2011) studied the vertebral levels of the origins of the CT and SMA in 62 cadavers and found 15 cases where the CT originated at the level of the upper third of the first lumbar vertebra, while in 21 cases the SMA origin was at the level of the middle third of the first lumbar vertebra [5]. In 4/62 cases these authors found a high origin of the CT at the level of the 10th thoracic vertebra [5].

Ekingen et al. studied more than 200 MDTC angiograms [4]. Unlike our study, they found additional vertebral levels as landmarks. For the CT they found 7/238 cases (2.94%) with the CT origin at the level of the T11/T12 intervertebral disc and 1/238 cases (0.42%) with the aortic origin at the level of the L1/L2 disc [4]. Additional levels were found also for the SMA: 11/257 cases (4.28%) at the level of the middle 3rd of the 12th thoracic vertebra and 1/257 cases (0.39%) at the level of the middle 3rd of the 2nd lumbar vertebra [4]. Ekingen et al. found that the distance measurements between the CT–SMA were statistically significant [4]. None of these studies [1,2,3,4] evaluated the combined patterns of the vertebral origins of the CT and SMA. Mirjalili et al. documented in adults the vertebral levels of origins for the CT and SMA [28]. They found that the vertebral level of the CT was most commonly at T12 (42%) but varied between the intervertebral disk between the 11th and the 12th thoracic vertebrae and the lower half of the 1st lumbar vertebra [28]. Mirjalili et al. found that the SMA origin was usually located at L1 (77%) and the range of variation was between the lower half of the 12th thoracic vertebra and the lower half of the 2nd lumbar vertebra [28]. These authors did not use a grading score with three levels for each vertebra, nor did they consider the intervertebral disks’ levels when they referred the aortic origins of the CT and SMA to the vertebral column. Additionally, Mirjalili et al. (2012) did not evaluate the CT and SMA’s combined patterns. It is therefore difficult to refer the present results to theirs.

Several studies only analyzed the CT vertebral level without SMA. Surucu et al. (2003) found the CT origin at the level of the 12th thoracic vertebra in 79.8% of cases, at the 1st lumbar vertebra in 14.4% and the level of the 11th thoracic vertebra in 3.8% of cases [29]. We did not find a T11 level of the CT origin in this study. Pinal-Garcia et al. (2018) conducted a cadaver study and found that, in 90% of cases, the CT aortic origin was “between the 12th thoracic and 1st lumbar vertebral bodies” [30]. Venieratos et al. (2013) found the vertebral levels of origin of the CT ranged from the middle 3rd of the 10th thoracic vertebra to the intermediate 3rd of the 2nd lumbar vertebra, the median level being at the upper 3rd of the 1st lumbar vertebra [31]. A recent study by Juszczak et al. (2020), performed on 50 cadavers found that the CT’s level of origin was at the intervertebral disc between T12 and L1 in all the cases [32].

## 5. Limitations

Our study did not evaluate the impact of differently sized and shaped vertebrae on the origin of the CT/SMA. There are no details in the literature regarding this possibility. Even if this would be extremely important, from a surgical point of view, it would have required a significantly larger study group, able to yield potentially significant correlations. Moreover, in such studies, including the present one, the variable vertical pattern of the CT and SMA aortic origins was not correlated with further anatomical variations of the CT and SMA branches.

## 6. Conclusions

This wide variability of the origins of the CT/SMA types emphasizes the need for surgeons to evaluate them on a case-by-case basis rather than using a general anatomical pattern as a reference. As visualization of the surgical field might be limited, preoperative knowledge of variant anatomy could help to adequately plan the surgery, as considered previously [21]. Use of computed tomography during preoperative planning could help or avoid meticulous surgical dissection within the surgical field.

## Figures and Tables

**Table 1:** The vertebral levels (types 1–9) of the aortic origins of the CT and SMA in males (*n* = 68) and females (*n*’ = 39).

Type	Males		Females	
	CT Origin	SMA Origin	CT Origin	SMA Origin
1	5 (7.35%)		2 (5.12%)	
2	6 (8.82%)		4 (10.25%)	
3	12 (17.64%)	1 (1.4%)	8 (20.51%)	2 (5.12%)
4	10 (14.7%)	9 (13.23%)	11 (28.2%)	2 (5.12%)
5	18 (26.47%)	5 (7.35%)	8 (20.51%)	7 (17.94%)
6	13 (19.11%)	15 (22.05%)	3 (7.69%)	12 (30.76%)
7	4 (5.88%)	24 (35.29%)	3 (7.69%)	11 (28.2%)
8		10 (14.7%)		4 (10.25%)
9		4 (5.88%)		1 (2.56%)

**Table 2:** Combinations of vertebral levels of origins of the celiac trunk (CT) and superior mesenteric artery (SMA).

Combined Types CT/SMA	Males (*n* = 68)	Females (*n*’ = 39)	Total Cases (*n* = 107)
1/3	1	2	3
1/4	2	0	2
1/6	1	0	1
1/7	1	0	1
2/4	4	1	5
2/5	0	1	1
2/6	1	1	2
2/7	1	1	2
3/4	4	2	6
3/5	5	4	9
3/6	3	1	4
3/7	0	1	1
4/5	0	2	2
4/6	5	8	13
4/7	3	0	3
4/8	2	1	3
5/6	2	2	4
5/7	13	6	19
5/8	3	1	4
6/6	2	0	2
6/7	6	2	8
6/8	4	1	5
6/9	1	0	1
7/7	0	0	0
7/8	1	1	2
7/9	3	1	4

**Table 3:** CT type depending on the gender of the subject.

	CT Type							
Gender	1	2	3	4	5	6	7	Total
Male	5	6	12	10	18	13	4	68
Female	2	4	8	11	9	3	2	39
Total	7	10	20	21	27	16	6	107

**Table 4:** SMA type depending on the gender of the subject.

	SMA Type							
Gender	3	4	5	6	7	8	9	Total
Male	1	10	5	14	24	10	4	68
Female	2	3	7	12	10	4	1	39
Total	3	13	12	26	34	14	5	107

**Table 5:** Contingency table of the associations/combinations of CT and SMA types.

	SMA Type							
CT Type	3	4	5	6	7	8	9	Total
**1**	3	2	0	1	1	0	0	7
**2**	0	5	1	2	2	0	0	10
**3**	0	6	9	4	1	0	0	20
**4**	0	0	2	13	3	3	0	21
**5**	0	0	0	4	19	4	0	27
**6**	0	0	0	2	8	5	1	16
**7**	0	0	0	0	0	2	4	6
**Total**	3	13	12	26	34	14	5	107
